# Evidence of increased cardiovascular disease risk in left-handed individuals

**DOI:** 10.3389/fcvm.2023.1326686

**Published:** 2023-12-14

**Authors:** Abigayle B. Simon, Kimberly Norland, Marsha Blackburn, Sumang Zhao, Xiaoling Wang, Ryan A. Harris

**Affiliations:** Georgia Prevention Institute, Medical College of Georgia, Augusta University, Augusta, GA, United States

**Keywords:** handedness, endothelial function, heart rate variability, cardiovascular disease, screening

## Abstract

**Background:**

Approximately 10% of the world is left-handed (LH). Research suggests that LH individuals may have shorter lifespans compared to right-handed (RH) individuals. LH individuals also appear to have more cardiovascular disease (CVD) related conditions like diabetes and cancer. Thus, the present study sought to test the hypothesis that vascular function and heart rate variability (HRV), both key indicators of CVD risk, would be lower in LH compared to RH individuals.

**Methods:**

Three hundred seventy-nine participants, 18–50 years old, were enrolled. Flow-mediated dilation (FMD), a bioassay of vascular endothelial function and standard deviation of R-R interval (SDNN), a parameter of HRV, were evaluated as indices of CVD risk. Data are reported as mean ± SD.

**Results:**

12.1% of the participants were LH. No differences in demographics or clinical laboratory values were observed between groups, except high-density lipoprotein (HDL) was higher (*p *= 0.033) in RH. FMD was significantly (*p *= 0.043) lower in LH (6.1% ± 3.2%) compared to RH (7.6% ± 3.8%), independent of age, sex, race, BMI, and HDL. Total power (*p *= 0.024) and low-frequency power (*p *= 0.003) were lower in LH compared to RH. Additionally, SDNN was lower (*p *= 0.041) in LH (47.4 ± 18.8 ms) compared to RH (54.7 ± 22.3 ms). A negative correlation between FMD and mean arterial pressure (*r *= −0.517; *p *< 0.001) was observed in LH; no relationships were observed in RH (all *p *> 0.05).

**Conclusion:**

Vascular endothelial function and HRV are lower in LH compared to RH. In addition, relationships between FMD and traditional CVD risk factors were only observed in LH. These data support an increased risk of CVD in LH.

## 1. Introduction

Handedness is characterized by the preference for using one hand over the other and represents a distinctive, but multifaceted field of study. Handedness is influenced by a combination of genetic and environmental factors. In fact, the heritability of handedness only equates to ∼25% (1), which indicates that factors other than genetics are certainly involved in developing dominant use of one hand over the other. Roughly 90% of individuals are right-handed (RH), and this high prevalence has been observed consistently throughout the world (2). Accordingly, and perhaps unsurprising, the contemporary world is predominantly tailored to accommodate RH individuals in various aspects, such as the design of scissors, keyboards, and buttons on cameras.

Although the clinical significance of handedness has yet to be fully elucidated, there is a paucity of data to support that handedness may negatively affect an individual's health. Individuals who are left-handed (LH) exhibit a staggering 9-year decrease in lifespan compared to individuals who are RH (3). In addition, the prevalence of various comorbidities of cardiovascular disease (CVD), including diabetes (4), cancer (5), asthma (6), dyslexia (7), and migraines (8) are also higher in LH individuals compared to RH individuals. Moreover, LH patients with coronary artery disease are more likely to have a history of ventricular tachycardia and ventricular fibrillation compared to age-matched RH patients with coronary artery disease (9). Taken together, these data support an increased health burden in those who are LH; however, the mechanisms have yet to be elucidated.

A significant association between abnormal cardiac autonomic function and left-handedness has also been observed. An abnormal QRS-T angle on electrocardiogram, an indicator of abnormal ventricular repolarization of the heart, has been observed more frequently in LH individuals and increases the potential for arrhythmias or sudden cardiac death (10). Additionally, a single report has demonstrated that heart rate variability (HRV), an indicator of autonomic nervous system function, is lower in LH individuals compared to their RH counterparts (11).

Within the intricate landscape of human genetics and overall health and well-being, an interesting question persists, whether handedness, a seemingly innocent trait, could be linked to greater risk of CVD. A lower HRV is an established, independent risk factor for increased CVD risk (11), and the flow-mediated dilation (FMD) test is a bioassay of nitric oxide (NO) bioavailability and non-invasive assessment of vascular endothelial function (12). Endothelial dysfunction precedes the development of atherosclerotic CVD, and the FMD test is also an independent predictor of future CVD risk and events (12), beyond traditional risk factors (13). Accordingly, the present study sought to test the hypothesis that both FMD and HRV, independent risk factors of CVD, would be lower in individuals who were LH compared to their RH counterparts.

## 2. Methods

### 2.1. Experimental design

All participants arrived to the Georgia Prevention Institute at Augusta University following an overnight fast and having abstained from alcohol, tobacco, and moderate exercise for the prior 12 h. The visit consisted of an informed consent process, body composition measurements, blood pressures, and anthropometric measures. A single stick blood draw was performed to assess a lipid panel and determine glycated hemoglobin (HbA_1c_) (Laboratory Corporation of America Holdings, Birmingham, AL) to evaluate metabolic and glycemic status, respectively. Height and weight were determined using a stadiometer and standard platform scale (CN20, DETECTO, Webb City, MO), respectively, and were utilized to calculate body mass index (BMI). Total body fat was determined using dual-energy x-ray absorptiometry (QDR-4500W; Hologic, Marlborough, MA).

### 2.2. Participant characteristics

Participants were recruited as part of an ongoing, longitudinal twin cohort parent study. 379 individuals (159 twin pairs and 61 singletons; men: *n* = 147, women: *n* = 232) were recruited to participate in the current study between 2018 and 2022. Participants were excluded if they (1) were currently pregnant or nursing, (2) had a previous diagnosis of cardiovascular disease including myocardial infarction, congestive heart failure, or stroke, (3) had a previous diagnosis of lung disease including chronic obstructive pulmonary disease, or (4) had a previous diagnosis of cancer. All study protocols were approved by the Institutional Review Board at Augusta University.

### 2.3. Heart rate variability

Short-term time- and frequency-domain heart rate variability parameters were measured in most participants using the Finapres Nova Basic (Finapres Medical Systems, Netherlands) for at least 256 cardiac cycles (R-R intervals). Participants laid in a supine position for at least 10 min prior to measurement collection. The average measurement period was 5 min. A filter was applied to detect and remove abnormal beats by the system, which calculated the heart rate variability parameters based on the HRV guidelines (14). The power spectral analysis applied a Hanning window to the signals, and a Fourier transformation of the R-R intervals was performed. The time-domain variables used were the standard deviation (SD) of normal R-R intervals (SDNN) and the root-mean-square of successive differences in R-R intervals (RMSSD). Frequency-domain variables included high-frequency (HF) power (0.15–0.40 Hz), low-frequency (LF) power (0.04–0.15 Hz), very-low frequency (VLF) power (0.0033–0.04 Hz), total power (TP), and LF power/HF power ratio. The HF band, also known as the respiratory band, is indicative of parasympathetic nervous system activity as it corresponds to the variations in heart rate that are mediated by the respiratory cycle (11). The LF band, also known as the baroreceptor band, is a combined influence of both the sympathetic and parasympathetic nervous system activity on heart rate modulation (15). The VLF band represents sympathetic nervous system activity and physiological stress (11). Lastly, total power represents the sum of the energy in the VLF, LF, and HF bands (11), and higher total power is generally associated with an enhanced overall autonomic equilibrium and cardiovascular health (16).

### 2.4. Vascular endothelial function

Endothelial-dependent vasodilation was determined using the brachial artery flow-mediated dilation test in accordance with published guidelines (12). Briefly, participants laid in a rested, supine position for at least 15 min to obtain steady blood flow and ensure a hemodynamically stable state following the HRV assessment. A 12-MHz linear probe transducer was held above the antecubital fossa and simultaneous B-mode and blood velocity profiles (duplex mode) of the brachial artery using a Doppler ultrasound (Loqic 7: General Electric Company, Milwaukee, WI) were obtained. Baseline data was collected for 30 s. Subsequently, the forearm occlusion cuff (E-20 rapid cuff inflator; D.E. Hokanson) that was placed immediately distal to the medial epicondyle was rapidly inflated to 250 mm Hg. Following 5 min of forearm occlusion, the cuff was released, and brachial artery diameter and blood velocity were continuously recorded for 2 min. R-wave gating (Accusync 72, Accusync Medical Research Corporation, Milford, CN) was utilized to capture end-diastolic arterial diameters for automated offline analysis of brachial artery vasodilation (Medical Imaging Applications, Coralville, Iowa). FMD (%) is reported as the percent of the maximal brachial artery dilation diameter from baseline diameter. Cumulative shear rate (s^−1^, area under the curve, AUC) was determined using the trapezoidal rule, every 4 s for the first 20 s following cuff release, and every 5 s thereafter for the remainder of the 2-min data collection period.

### 2.5. Statistical analyses

All analyses were performed using SPSS Statistics Version 28. A Pearson Chi-Square test was performed to identify any differences in the proportion of men vs. women (sex) and non-Hispanic Black people vs. non-Hispanic White people (race) between LH and RH individuals. In order to control for the dependence of twin pairs, generalized estimating equations (GEE) were performed to identify group differences (i.e., LH vs. RH) in demographics and clinical laboratory markers. GEE is a multiple regression technique that allows for non-independence of twin or family data yielding unbiased standard errors and *p*-values (17). GEE was also used to determine differences in FMD parameters and HRV parameters between LH and RH individuals. A Pearson's Correlation was used to assess relationships among traditional CVD risk factors [i.e., mean arterial pressure (MAP)] and both FMD and SDNN. Data are reported as mean ± standard deviation (SD) unless otherwise noted. Statistical significance (*) was set at *p* < 0.05.

## 3. Results

### 3.1. Participants

Participant demographics and clinical laboratory values are presented in Table 1. 12.1% of the participants were LH, which is consistent with the proportion of left-handedness worldwide. A similar proportion of men and women were enrolled between LH and RH groups (*p *= 0.308). In addition, a similar proportion of non-Hispanic Black people and non-Hispanic White people were also enrolled between groups (*p *= 0.075). Individuals within the RH group had significantly (*p *= 0.033) higher high-density lipoprotein (HDL) compared to those in the LH group. No differences (all *p *> 0.05) in any other participant demographics or clinical laboratory values were observed between groups.

**Table 1:** Participant characteristics.

Variable	Overall (*n* = 379)	Left-handed (*n* = 46)	Right-handed (*n* = 333)	*p*-value
Age (year)	35 ± 6	34 ± 7	35 ± 6	0.053
Height (cm)	170 ± 10	171 ± 8	170 ± 10	0.913
Weight (kg)	89 ± 29	93 ± 30	89 ± 29	0.458
BMI (kg/m^2^)	30.8 ± 8.7	31.8 ± 9.3	30.6 ± 8.6	0.246
Body fat (%)	36.6 ± 9.7	35.6 ± 9.6	36.8 ± 9.7	0.668
SBP (mm Hg)	23 ± 13	123 ± 14	123 ± 13	0.789
DBP (mm Hg)	77 ± 10	76 ± 10	77 ± 9	0.645
HbA1c (%)	5.5 ± 0.6	5.6 ± 0.9	5.5 ± 0.6	0.106
TC (mg/dl)	178 ± 35	174 ± 34	179 ± 35	0.325
HDL (mg/dl)	53 ± 15	49 ± 13	54 ± 15	**0**.**033**
LDL (mg/dl)	106 ± 32	102 ± 29	106 ± 32	0.652
TRIG (mg/dl)	102 ± 74	119 ± 117	100 ± 66	0.397

BMI, body mass index; HbA1c, hemoglobin A1c; TC, total cholesterol; HDL, high-density lipoprotein; LDL, low-density lipoprotein; TRIG, triglycerides.

Data are presented as mean ± SD; General estimating equations. Bold values indicate statistical significance.

### 3.2. Indices of cardiovascular disease risk and handedness

#### 3.2.1. Flow-mediated dilation

**Figure 1:**
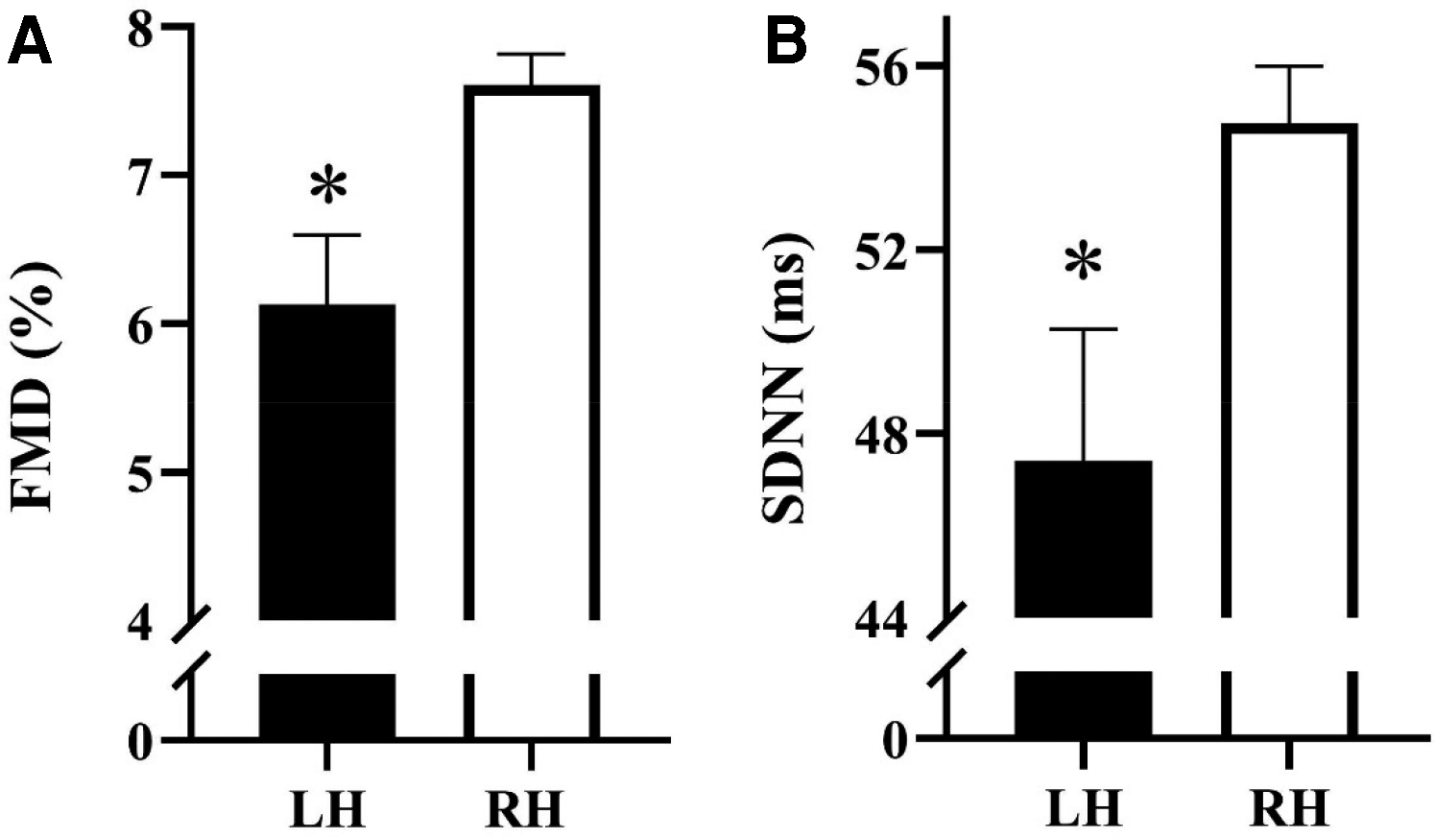
(**A**) Flow-mediated dilation (FMD) between left-handed (LH) and right-handed (RH) individuals; *n* = 379 (LH = 46, RH = 333) and (**B**) SDNN between LH and RH individuals; *n* = 359 (LH = 43, RH = 316). Data are presented as mean ± SEM. *indicates a significant difference from RH.

The parameters of the FMD test in LH vs. RH individuals are presented in Table 2. There were no differences (all *p *> 0.05) in baseline diameter, peak diameter, and shear rate between groups. Figure 1A illustrates FMD in LH individuals vs. RH individuals. FMD was significantly (*p *= 0.043) lower in LH compared to RH participants, independent of age, sex, race, BMI, and HDL. No sex-based differences were observed between groups (all *p *> 0.05).

**Table 2:** Parameters of the FMD test and indices of HRV.

Variable	Overall (*n* = 379)	Left-handed (*n* = 46)	Right-handed (*n* = 333)	*p*-value
Endothelial function				
FMD baseline diameter (cm)	0.338 ± 0.065	0.351 ± 0.054	0.337 ± 0.067	0.346
FMD peak diameter (cm)	0.362 ± 0.062	0.372 ± 0.051	0.361 ± 0.065	0.482
FMD shear rate (s^−1^, AUC)	43,662 ± 18,722	38,447 ± 15,013	44,382 ± 19,085	0.102
Heart rate variability				
RMSSD (ms)	46.0 ± 22.6	42.7 ± 21.8	46.5 ± 22.6	0.156
HR (bpm)	67 ± 10	68 ± 10	67 ± 10	0.524
TP (ms^2^)	2,714 ± 2,383	1,881 ± 1,519	2,827 ± 2,458	**0**.**024**
VLF power (ms^2^)	941 ± 944	697 ± 750	975 ± 964	0.114
LF power (ms^2^)	759 ± 663	481 ± 377	797 ± 684	**0**.**003**
HF power (ms^2^)	588 ± 536	528 ± 454	596 ± 547	0.249
LF/HF ratio	1.86 ± 1.70	1.53 ± 1.38	1.91 ± 1.73	0.194

RMSSD, root-mean-square of successive RR interval differences; HR, heart rate; TP, total power; VLF, very-low frequency; LF, low-frequency; HF, high-frequency.

Data are presented as mean ± SD; Generalized estimating equations. Bold values indicate statistical significance.

#### 3.2.2. Heart rate variability

The average ectopic beat frequency for the overall, LH, and RH groups, were 0.12% ± 0.92%, 0.08% ± 0.33%, and 0.13% ± 0.97%, respectively. Indices of heart rate variability are presented in Table 2. Total power (*p *= 0.024) and LF power (*p *= 0.003) were lower in LH compared to RH. There were no other differences (all *p *> 0.05) in HRV parameters between groups. Figure 1B illustrates SDNN in LH vs. RH individuals. LH individuals exhibited a significantly (*p *= 0.041) lower SDNN compared to RH individuals, independent of age, sex, race, BMI, and HDL. No sex-based differences were observed between groups (all *p *> 0.05).

#### 3.2.3. Relationships between both FMD and SDNN with traditional risk factors for CVD

**Figure 2:**
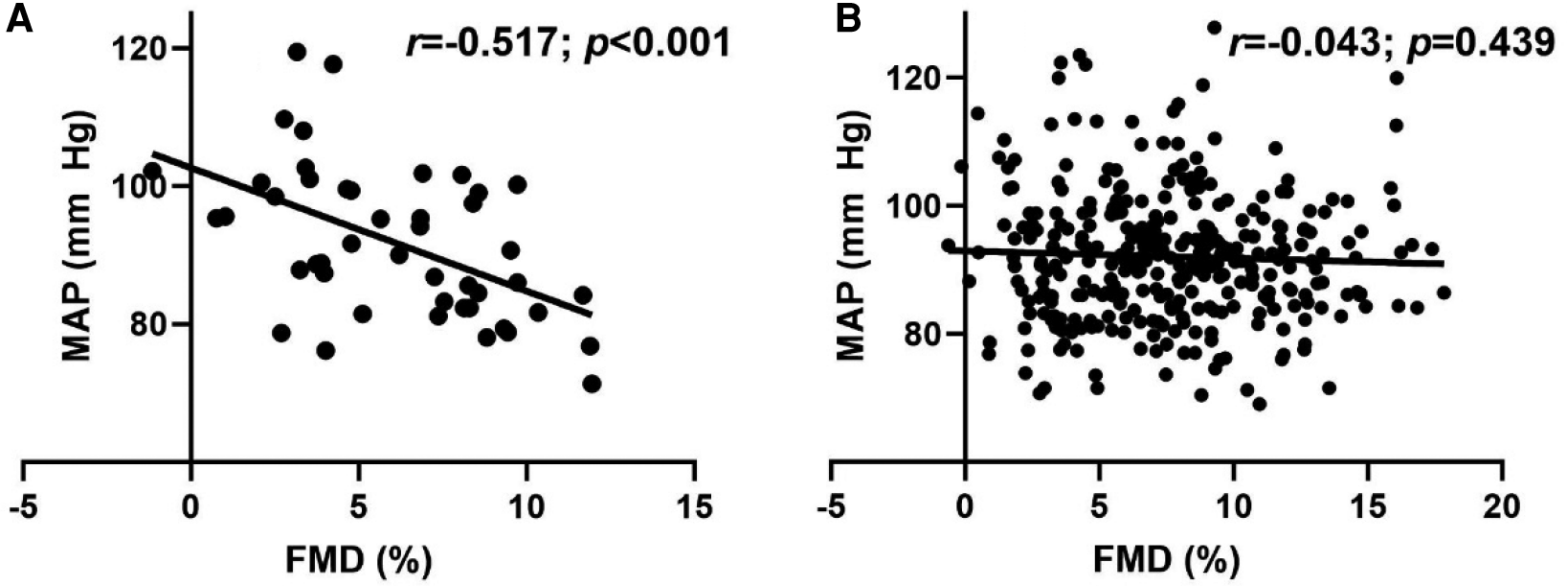
Relationship between flow-mediated dilation (FMD) and mean arterial pressure (MAP) in (**A**) left-handed (LH; *n* = 46) and (**B**) right-handed (RH; *n* = 333) individuals. Pearson's Correlation.

Overall, no relationship (*r *= −0.092; *p *= 0.073) was observed between MAP and FMD. Figure 2, however, illustrates the significant inverse relationships between MAP and FMD (Figure 2A; *r *= −0.517; *p *< 0.001) in LH, but not RH (Figure 2B; *r *= −0.043; *p *= 0.439).

**Figure 3:**
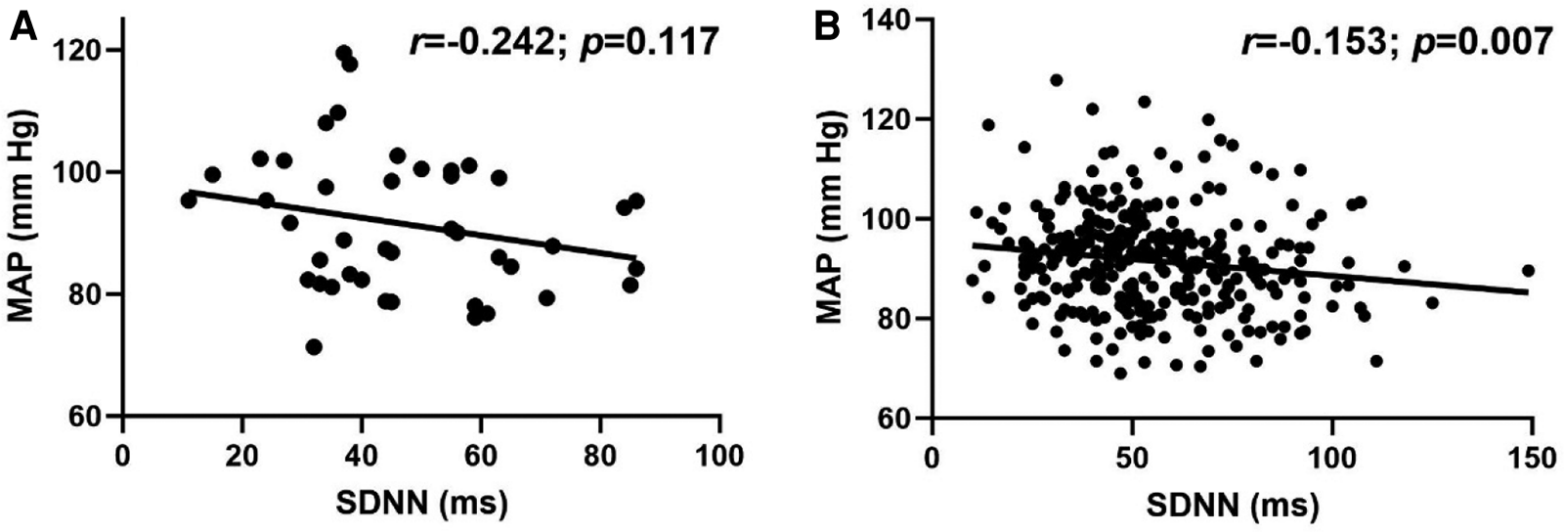
Relationship between standard deviation of R-R interval (SDNN) and mean arterial pressure (MAP) in (**A**) left-handed (LH; *n* = 43) and (**B**) right-handed (RH; *n* = 316) individuals. Pearson's Correlation.

Overall, there was a modest, but significant inverse relationship between MAP and SDNN (*r *= −0.160; *p *= 0.002). Although the association between MAP and SDNN was not significant in LH individuals (Figure 3A; *r *= −0.242; *p *= 0.117), the relationship was significant in RH individuals (Figure 3B; *r *= −0.153; *p *= 0.007).

## 4. Discussion

Existing data lends support to adverse health consequences in LH individuals, particularly with a decreased life expectancy and an overall increased prevalence of various health conditions in LH compared to RH individuals (4–8). The present study is the first to report that flow-mediated dilation is decreased in apparently healthy LH individuals compared to their RH counterparts, a finding that persisted after controlling for age, sex, race, BMI, and HDL. In addition, autonomic nervous system dysfunction was observed in LH individuals compared to RH individuals. Furthermore, significant negative relationships between MAP with FMD were only observed in the LH group. Collectively, findings from the present investigation demonstrate that LH individuals have an increased risk of CVD compared with their RH counterparts.

### 4.1. Handedness and vascular endothelial function

Endothelial dysfunction plays a pivotal role in the etiology of atherosclerotic CVD (12). The FMD test is a non-invasive assessment of vascular endothelial function in humans and represents an index of NO bioavailability (12). Perhaps most important, the assessment of FMD can predict future CVD risk and CVD events (13). The present findings demonstrate that LH individuals exhibit a lower FMD compared to RH individuals. Given that a 1% decrease in FMD is equivalent to a 9% increase in the future risk of CVD (18), these data would suggest that LH individuals have a ∼14% increased risk of CVD compared to those who are RH. Although the shorter longevity observed in LH individuals (3) was recently questioned by a modeling study that takes into account social historical trend (19), the reported decreased survival in LH may be mediated by an increase in CVD risk that is observed in the present investigation. Nonetheless, further research is certainly needed to determine the cardiovascular consequences of being LH.

It is widely recognized that hypertension is a traditional risk factor that contributes to an increase in CVD within the general population. Although there were no differences in MAP between groups in the present study, significant inverse relationships between FMD and MAP were only identified in LH individuals. It is important to note that the cohort that participated in the present investigation was relatively young with no overt CVD. Nonetheless, these findings underscore the sensitivity of slight fluctuations in blood pressure as they related to FMD, specifically in LH individuals. These findings are the first to document that handedness, specifically left-handedness, may represent a novel risk factor that may be associated with the development of future CVD. Although the present investigation cannot infer cause and effect, further investigations into the potential mechanisms responsible for how handedness increases CVD risk are warranted. Moreover, the present findings emphasize the importance of personalized medicine. Indeed, vigilant monitoring for changes in traditional CVD risk factors may be required, as these minor alterations in traditional risk factors could impact future cardiovascular health disproportionately based on handedness.

### 4.2. Handedness and heart rate variability: time domain assessment

Heart rate variability is used as a non-invasive method to assess autonomic nervous system functionality. This methodological assessment of heart rate can provide insight into the balance of the sympathetic and parasympathetic nervous system, representing an index of physiological homeostasis (20). In general, it has been well established that a lower HRV is associated with a greater risk of cardiovascular events and overall mortality (21–23). Specific to handedness, a significantly lower cardiac autonomic function has been observed in LH compared to RH individuals. Interestingly and important to note; however, this previous investigation was conducted only in men (10). In a separate study conducted in young adults, LH individuals exhibited an imbalanced autonomic system compared to RH individuals (24). In the present investigation, SDNN, a global index of HRV and predictor of CVD risk (11, 25), was significantly lower in LH compared to RH, and no sex-based differences were observed in our cohort of middle-aged adults. Lower SDNN values represent more compromised health (11), and values below 50 ms are indicative of an unhealthy classification (25). Conversely, SDNN values exceeding 50 ms typically span the spectrum from moderately healthy to healthy classifications (14). Unhealthy classifications were observed in the present LH group, whereas RH individuals exhibited moderately healthy classifications. Overall, the HRV results of the current study align with the previous findings; however, the present investigation bridges a critical knowledge gap by encompassing both men and women and utilizing a sample size that was comprised of a relatively large LH cohort. Further, in the context of assessing autonomic nervous system function via HRV, root mean squared of successive differences between R-R intervals (RMSSD) was also considered. RMSSD captures short-term variations in heart rate that are primarily influenced by parasympathetic activity. It provides a more focused perspective compared to the broader variability assessed by SDNN (11). The differences in RMSSD values observed between LH (42.7 ± 21.8 ms) and RH (46.4 ± 22.6 ms) in the present study, although trending in the same direction, were not statistically significant (*p *= 0.156). SDNN and RMSSD can effectively serve as interchangeable proxies for each other (26); however, disparate findings between SDNN and RMSSD have also previously been reported (27, 28). Importantly, RMSSD was lower in LH compared to RH, which is indicative of a greater autonomic nervous system dysfunction (11) and represents an increase in CVD risk. Further, although the overall relationship between SDNN and MAP in RH individuals was statistically significant, they only explain ∼3% of the variance. These data are in line with previous literature (29, 30), and these clinically insignificant findings are likely due to the young age of the investigated cohort. Indeed, more robust relationships between SDNN and both MAP have been reported, particularly in older populations and individuals with hypertension (31). Accordingly, further research in diverse age groups and clinical contexts may provide a more comprehensive understanding of these associations, and investigation into aging and handedness is certainly warranted.

### 4.3. Handedness and heart rate variability: frequency-domain assessment

Within the analysis of HRV, the present investigation delves into frequency-domain assessments that offer valuable insights into the dynamics of the autonomic nervous system and the equilibrium between sympathetic and parasympathetic activities (11). Specifically, greater total power often indicates a more adaptable autonomic nervous system capable of responding effectively to various physiological and psychological challenges. The present investigation is the first to demonstrate that total power was significantly lower in LH individuals, which suggests that their autonomic nervous system may not possess the same level of adaptability as observed in RH individuals. Indeed, impaired adaptability within the autonomic nervous system carries substantial clinical significance, given its potential to contribute to a spectrum of cardiovascular disorders, including but not limited to hypertension, arrhythmias, and heart failure (32). A reduced LF power has been shown to predict sudden cardiac death in patients with congestive heart failure (33). Interestingly, LF power was significantly lower, and HF power was higher in LH compared to RH individuals (Table 2). Accordingly, these data would suggest that RH individuals have greater baroreceptor/parasympathetic dominance and lower respiratory/sympathetic dominance compared to their LH counterparts, albeit a statistically similar LF/HF ratio. Given that this ratio was originally based on 24 h recordings (34) and does not correlate well with short term values, the individual frequency data can be interpreted in isolation without interpretation confusion of the ratio. Nonetheless, these data support that compared with RH, LH individuals exhibit an impairment in dynamic autonomic regulation that is important for cardiovascular health (23).

### 4.4. Experimental considerations

The present investigation aimed to determine differences in vascular endothelial function and HRV between RH and LH individuals. The FMD test is typically assessed in the right arm, independent of handedness. Although the present study did not assess FMD in both arms, there are only two small studies, with conflicting results, that have evaluated FMD between arms. Utilizing the recommendations on performing the FMD test (12), no differences in FMD have been observed between arms in both men and women (35). In contrast, a study conducted in only 13 right-hand dominant men documented distinct differences in FMD between arms. Interestingly, however, no relationship in FMD between the dominant and non-dominant arms was observed (36). In addition, inter-arm blood pressure is consistent over time, regardless of handedness, suggesting that measuring blood pressure in both arms may not be necessary (37). Furthermore, assessment of arterial stiffness using pulse wave velocity in both the right and left sides (both arms and ankles) were found to be reproducible (38). It is important to note that the clinical significance of the FMD test was determined by performing FMD in the right arm, which is consistent with the methods used in the present investigation. Nonetheless, results of previous investigations, coupled with the current findings, support the clear need for future research to explore these inter-arm differences in FMD, particularly in the context of handedness. In addition, it is important to note that 24-h and short-term HRV measurements are not interchangeable. With that being said, both short and long-term recordings are effective at assessing autonomic nervous system function in humans (11) and should not underscore the observation that LH individuals exhibited a lower SDNN compared to their RH counterparts.

In conclusion, for the first time, the present investigation demonstrates that LH individuals exhibit lower vascular endothelial function compared to their RH counterparts. In addition, present findings concur with previous reports that demonstrate a reduced baroreflex sensitivity and parasympathetic dominance in LH compared to RH individuals. Moreover, the observed inverse relationships between FMD and MAP emphasize the fact that handedness may represent an independent risk factor for CVD and warrant earlier evaluation of traditional CVD risk factors, particularly in LH individuals.

## Data Availability

The raw data supporting the conclusions of this article will be made available by the authors, upon reasonable request.
